# MBB-MOGWO: Modified Boltzmann-Based Multi-Objective Grey Wolf Optimizer

**DOI:** 10.3390/s24051502

**Published:** 2024-02-26

**Authors:** Jing Liu, Zhentian Liu, Yang Wu, Keqin Li

**Affiliations:** 1 College of Computer Science, Inner Mongolia University, Hohhot 010021, China; liujing@imu.edu.cn (J.L.); liuzhentian@cmhi.chinamobile.com (Z.L.); 22109007@mail.imu.edu.cn (Y.W.); 2 Department of Computer Science, State University of New York, New Paltz, NY 12561, USA

**Keywords:** Boltzmann selection, multi-objective grey wolf optimizer, optimization algorithm, web service composition

## Abstract

The primary objective of multi-objective optimization techniques is to identify optimal solutions within the context of conflicting objective functions. While the multi-objective gray wolf optimization (MOGWO) algorithm has been widely adopted for its superior performance in solving multi-objective optimization problems, it tends to encounter challenges such as local optima and slow convergence in the later stages of optimization. To address these issues, we propose a Modified Boltzmann-Based MOGWO, referred to as MBB-MOGWO. The performance of the proposed algorithm is evaluated on multiple multi-objective test functions. Experimental results demonstrate that MBB-MOGWO exhibits rapid convergence and a reduced likelihood of being trapped in local optima. Furthermore, in the context of the Internet of Things (IoT), the quality of web service composition significantly impacts complexities related to sensor resource scheduling. To showcase the optimization capabilities of MBB-MOGWO in real-world scenarios, the algorithm is applied to address a Multi-Objective Problem (MOP) within the domain of web service composition, utilizing real data records from the QWS dataset. Comparative analyses with four representative algorithms reveal distinct advantages of our MBB-MOGWO-based method, particularly in terms of solution precision for web service composition. The solutions obtained through our method demonstrate higher fitness and improved service quality.

## 1. Introduction

The Multi-Objective Optimization Problem (MOP) is widely used to address common problems in the fields of economics, engineering, and the Internet of Things (IoT). In the context of IoT with numerous sensors, related studies primarily focus on tasks such as minimizing request response time and energy consumption [1], developing optimal scheduling strategies to conserve energy [2], and identifying malicious traffic. These tasks require a comprehensive consideration of factors and objectives such as service response time, workload, and energy consumption of each sensor in IoT. However, these objectives conflict with each other. Therefore, solving the optimization problem under multi-objective tasks is a critical issue in the current IoT and other important tasks. In contrast, single-objective optimization usually focuses on just one objective function, so the optimal value for such a function could be obtained by the best solution. MOP considers two or more objectives that are usually in conflict; that is, the improvement of one objective may bring negative effects to other objectives with a very high probability. Thus, equally optimal solutions should be computed to pursue the trade-off situation among all of the objectives, which is the Pareto Optimal Set (PS).

In the realm of multi-objective optimization problems (MOPs), the complexity of the solution set poses challenges for precise algorithms [3]. Conventional approaches struggle to effectively handle MOPs, prompting the exploration of heuristic [4] and meta-heuristic algorithms [5] for improved performance. Notable examples include Genetic Algorithm (GA), Ant Colony Optimization (ACO), Particle Swarm Optimization (PSO), Fruit Fly Optimization (FOA), and Differential Evolution (DE). Recently, the Multi-Objective Grey Wolf Optimizer (MOGWO) [6] has emerged as a promising swarm intelligence algorithm, building upon the foundation of the Grey Wolf Optimizer (GWO). MOGWO distinguishes itself with a faster convergence speed compared to its counterparts. In its implementation, MOGWO employs a fixed-size external archive to retain non-dominated solutions, and a grid-based method evaluates the Pareto front throughout the optimization process. However, traditional MOGWO exhibits drawbacks such as slow convergence in the later stages, making it susceptible to the pitfall of local optima.

The traditional Multi-Objective Grey Wolf Optimizer (MOGWO) algorithm consists of three primary steps: initializing the wolf pack, updating the position of the leader wolf and the entire wolf pack, and obtaining the solution set through continuous iteration. MOGWO exhibits a significant level of randomness during the wolf pack initialization. The algorithm’s search strategy predominantly relies on the values of *a* and |A| in the iteration process, both of which decrease as the number of iterations progresses. This tendency makes the algorithm prone to falling into local optima. Consequently, there is a need to optimize the search strategy in the MOGWO algorithm, aiming to formulate a more effective parameter update rule. Simultaneously, the position of the leading wolf holds crucial importance for the convergence of the MOGWO algorithm. Therefore, the primary research focus of this paper encompasses optimizing both the search strategy of the MOGWO algorithm and the selection strategy for the leading wolf. In this paper, a Modified Boltzmann-Based MOGWO is proposed, named MBB-MOGWO. As an optimized version of MOGWO, our MBB-MOGWO modifies the convergence factor used in the position update of a wolf into the variation of cosine law, and adopts Boltzmann selection strategy to get a better balance in the exploration and exploitation phase. We use multi-objective benchmark functions in CEC2009 [7] and ZDT [8] to perform the experimental evaluation. MBB-MOGWO is compared with four representative algorithms, i.e., MOGWO, NSGA-II, MOPSO, and MOEA/D. The experiment results illustrate that our MBB-MOGWO method overcomes the flaws in the traditional algorithms, that is, after those two major aspects of optimizations, MBB-MOGWO executes in fast convergence and well improves the precision of the solution, furthermore, it could not be trapped in the local optimum with a high probability.

Furthermore, within the realm of IoT, the web services composition can be leveraged to construct intricate intelligent systems with numerous sensors. Through the composition of services encompassing sensor data collection, device control, and user management, functionalities such as smart home automation and remote control can be effectively realized. To demonstrate the optimization effects of the MBB-MOGWO for solving actual optimization problems, it is further applied to deal with the MOP in the scenario of composing web service components. The MOP in web service composition-related studies is a non-linear and high-dimensional problem. Web service system is a platform-independent, low-coupling, and programmable-based software application [9]. The limitations of traditional single services in meeting the demands of complex tasks have prompted the emergence of web service compositions. Therefore, it is significant for a web service system that assembles the existing web services to build a powerful value-added service. The number of existing services is increasing rapidly. Many services have similar functions but different service quality attributes, or there are conflicts between services [10]. Therefore, it is a dilemma for users to select suitable web services for each subtask to make the whole web service system run optimally. Many related studies have proposed effective solutions for the web service composition problems, surveyed in [11,12,13,14]. So how to compute the optimal solution for this composition scenario is still very worth in-depth study nowadays. In this paper, we apply MBB-MOGWO to optimize the MOP issue in the web service composition problem. The real data records in the QWS dataset [15] are used to evaluate the composition effects. By comparing with four representative algorithms, our MBB-MOGWO-based method shows advantages in terms of the solution precision of web service composition.

The main contributions of this study are summarized as follows: (1) We propose a Modified Boltzmann-Based MOGWO to optimize the Wolf pack position update strategy in the MOGWO algorithm, so as to reduce the possibility of the algorithm falling into the local optimal and obtain better solutions. (2) We propose a new leader wolf selection strategy based on the Boltzmann selection strategy to improve the convergence speed of the algorithm. (3) The experimental results of several multi-objective test functions show that the proposed method is effective in terms of the quality and speed of the obtained solutions. We extend the method to the web service composition problem and verify the effectiveness of the algorithm.

The rest of our paper is organized as described below. Related work on MOGWO is discussed in Section 2. Section 3 presents the details about the MBB-MOGWO algorithm. Section 4 evaluates the performance of our MBB-MOGWO using common multi-objective benchmark functions. Section 5 integrates the MBB-MOGWO algorithm into the web service composition problem and evaluates its effects with the real data records in the QWS dataset. Finally, we conclude our work in Section 6 with future works.

## 2. Related Work

Compared with single-objective optimization, MOP tends to be more complex, which would consider two or more objectives and these objectives are usually in conflict. Therefore, corresponding multi-objective optimization algorithms need to be developed to optimize these objectives at the same time. Among the recently proposed algorithms, MOGWO is one of the most popular algorithms, with the advantage of a concise structure and fewer parameters to be adjusted. We will present a brief discussion of related work from these three categories in detail as follows.

### 2.1. Improved Initialization Population

In MOGWO, the first stage initializes population, after which the optimal set of solutions to the problem is obtained by stepwise iteration. Therefore, it is crucial for MOGWO to initializing the population. The quality of the initial population is quite important for global convergence speed, as well as the availability of solutions. A well-diversified initial population is beneficial to seek out the optimal solution. Luo et al. [16] were inspired by complex-valued encoding, which greatly expands individual information capacity. The genes of the gray wolf can be expressed as Equation (1):

(1) $$x_{p}=R_{p}+iI_{p},\;p=1,2,\cdots ,M$$

where $R_{p}$ and $I_{p}$ indicate the genes of gray wolves. The two variables are updated independently, thus enhancing the diversity of the population. Madhiarasan et al. [17] improved the traditional gray wolf population rank by dividing the gray wolf population into three groups, namely theta (θ), zeta (ζ) and psi (ψ). During the updating phase, the worst position in every group is thought over to minimizes the convergence time for better performance. Long et al. [18] introduced the good point set approach for improving the population initialization. When the same number is taken, the point sequences got by the good point set method would be distributed equably in whole feasible region, which get better diversity of individuals for population initialization.

In original MOGWO, the simple random initial method is used, but the method does not keep population diversity and converges to local optimum easily.

### 2.2. Improvements to the Search Mechanism

In the original MOGWO, *a* and *A* are used to regenerate the position for grey wolves. The value of parameter *a* decreases linearly, hence MOGWO has the weak capability of exploration and was easy to get into the dilemma of local optimum. Large randomness is only available when initializing the position of the grey wolves. Muangkote et al. [19] proposed that two different update strategies for grey wolves’ position are employed. A new strategy was introduced to calculate the distance vectors, i.e., randomly selected index values are used to update the vectors for improving randomness. Saremi et al. [20] presents the updating method for the grey wolves’ positions using the Evolutionary Population Dynamics (EPD). The worst individuals were removed in each iteration which were repositioned around the best solutions, such as α, β, δ or random position around the search space, which obtain better solutions. Malik et al. [21] showed Weighted distance Grey wolf optimizer which modified the original location update strategy of gray wolf, i.e., the weighted average for three positions is used as the new position instead of a simple average.

The exploration and exploitation phase in original MOGWO depends mainly on the |A|, while the update of the |A| depends on *a*. As |A| > 1, the MOGWO would search for the prey, which called the exploration phase. As |A| < 1, the MOGWO would pay close attention to the prey in search space, which called exploitation phase. Hence, |A| is one of the key factors to pursue optimal balance in exploration and exploitation. All the above papers improved the position update strategy, but the update strategy of |A| is not modified. Therefore, the algorithm is easy to enter exploitation phase and trap into the dilemma of local optimum. In our work, the update strategy of *a* is improved for extending the length of exploration phase and keeping away from local optimum, i.e., the using a nonlinear function to update *a*.

### 2.3. The Design of Hybrid Algorithms

Part of the literature focuses on integrating multiple algorithms to improve the MOGWO. Zhang et al. [22] presented a hybrid MOGWO with elite opposition, called EOGWO, where the elite opposition-based learning method was merged into GWO. Singh et al. [23] hybridized the Whale Optimizer Algorithm and Mean Grey Wolf Optimizer algorithm, named as HAGWO. In HAGWO, the spiral equation of the former algorithm was utilized to update the position of three leader wolves, which kept the balance in exploration and exploitation. Elgayyar et al. [24] used GWO algorithm to efficiently explore the search space and Bat swarm optimizer (BA) to refine the solution. Similarly, Zhang et al. [25] hybridized the Biogeography-Based Optimization (BBO) with GWO to fully utilize their advantages. Tawhid et al. [26] integrated genetic algorithm with GWO. To make the search solutions more diversified, the population was separated, together with using the genetic mutation operators towards the whole population. Through experimental results, the algorithm was effective for finding or approximating global minima. Similarly, Bouzary et al. [27] integrated a genetic algorithm and grey wolf optimizer algorithm, which was applied to service composition and optimal selection (SCOS) problems. Mirjalili et al. [28] proposed a MOGWO using decomposition, which cooperatively approximates the Pareto solution by defining the neighborhood relations between the scalarized subproblems decomposed by the multi-objective problem. In this paper, we combine the genetic algorithm and MOGWO, i.e., the Boltzmann selection is applied to select leader wolves.

## 3. MBB-MOGWO Design

Brief introduction and related formal definitions about MOP and MOGWO are firstly presented, and then the design details of our MBB-MOGWO is well explained.

### 3.1. Multi-Objective Optimization

The MOP is modeled where more than one objective to be optimized at the same time, and different objectives often conflict with each other. As we improve one objective, it is likely to cause the deteriorate of other objectives. Therefore, how to make multiple objectives optimized simultaneously and get a relatively better solution becomes a fatal problem. MOP could be described by the Formula (2) [29]:

(2) $$\begin{array}{cc} \; & minF\left(x\right)=\left(f_{1}\left(x\right),f_{2}\left(x\right),...,f_{m}\left(x\right)\right)\\ \; & s.t.\;x=\left(x_{1},x_{2},...,x_{n}\right)\in \Omega \end{array}$$

where Ω denotes the feasible domain of the search space; *x* denotes the *n*-dimensional decision variable in Ω; *m* denotes how many objectives should be optimized. The function *F* defines the mapping of *m* objective functions that to be optimized from the decision space Ω to the target space.

Unlike single-objective problems, multi-objective problems need to be compromised in multiple solutions, so that each target is close to the optimal solution. In most cases, a set of Pareto optimal solutions could be regarded as the solution of a specific MOP. The relevant definition is presented as follows.

**Definition** **1** (Dominant relationship)**.** Assume two vectors $x^{*}=\left[x_{1}^{*},x_{2}^{*},...,x_{D}^{*}\right]$ and $x=\left[x_{1},x_{2},...,x_{D}\right]$. If $\forall d\in \left[1,D\right],\;\mathrm{having}\;x_{d}^{*}\leq x_{d}\;\mathrm{and}\;\exists d_{0}\in \left[1,D\right],\;x_{d_{0}}^{*}<x_{d_{0}},\;\mathrm{then}\;x^{*}$ dominates x. Similarly, f(x) dominating f(y) should satisfy Formulas (3):

(3) $$\begin{array}{cc} \; & f_{i}\left(x\right)\leq f_{i}\left(y\right)\;\forall i\in \left[1,2,...,m\right]\\ \; & f_{j}\left(x\right)<f_{j}\left(y\right)\;\exists j\in \left[1,2,...,m\right] \end{array}$$

*That is, in the m objective functions, each objective function value of x is not greater than the objective function value of y, and at least one of them is smaller than y. f(x) dominates f(y) refers to f(x) is better than f(y).*

**Definition** **2** (Pareto optimal solution)**.** *Given a feasible point x, if not exists y∈S→f(y)<f(x), then x is the Pareto optimal solution of the specific MOP. That is, in the feasible domain space, there is no particle that can dominate the particle x, and we can also call it a non-dominated solution. Meanwhile, the Pareto Front is constructed from a set of all Pareto optimal solutions.*

### 3.2. An Overview of MOGWO

The MOGWO [6] is a kind of swarm intelligence algorithms that use the class system and group hunting within the grey wolf race. It has the features of strong convergence, few parameters, and easy implementation. The grey wolf has a formal hierarchy, that is, α, β, δ are the leaders in the wolves, and ω represents the group wolves. In MOGWO, each wolf in the wolves is regarded as a solution. The α represents the current optimal solution, the β represents the sub-optimal solution, and the δ represents the third-optimal solution. During the hunting phase, the wolves approach the food position (global optimal solution) with the lead of α, β, and δ. Through continuous exploration and exploitation, it would like to find the Pareto optimal solution for the MOP. Then we give the definitions of the MOGWO with a mathematical model.

**Definition** **3** (Relative distance)**.** *The relative distance between the grey wolf and the prey, as shown in Equation* (4):

(4) $$\begin{array}{cc} \; & D=\left|C\cdot X_{p}\left(t\right)-X\left(t\right)\right|\\ \; & C=2\cdot r_{1} \end{array}$$

*where the position of the prey is defined as $X_{p}$, and the position of a grey wolf is defined as X. t is the iteration number, and C is the synergy coefficient vector. $r_{1}$ is a random number which is assigned from [0,1].*

**Definition** **4** (Location update of a wolf)**.** *The position of each grey wolf is updated as searching for prey, as shown in Equation* (5):

(5) $$\begin{array}{cc} \; & X\left(t+1\right)=X_{p}\left(t\right)-A\cdot D\\ \; & A=2a\cdot r_{2}-a\\ \; & a=2-\frac{2}{MaxIt}t \end{array}$$

*where X indicates the updated position of a grey wolf. The convergence factor is represented as a and $r_{2}$ is the random number between [0,1]. MaxIt is the maximum number of iterations. From the equation, we can see that a decreases linearly from *2* to *0*.*

**Definition** **5** (Location update of wolves)**.** *The leader wolves guide wolves for searching and surrounding the prey (to find the global optimal solution) by updating their positions, as shown in Equation* (6):

(6) $$\begin{array}{cc} \; & D_{\alpha }=\left|C_{1}\cdot X_{\alpha }-X\right|\;X_{1}=X_{\alpha }-A\cdot D_{\alpha }\\ \; & D_{\beta }=\left|C_{2}\cdot X_{\beta }-X\right|\;X_{2}=X_{\beta }-A\cdot D_{\beta }\\ \; & D_{\delta }=\left|C_{3}\cdot X_{\delta }-X\right|\;X_{3}=X_{\delta }-A\cdot D_{\delta }\\ \; & X\left(t+1\right)=\frac{X_{1}+X_{2}+X_{3}}{3} \end{array}$$

**Figure 1:**
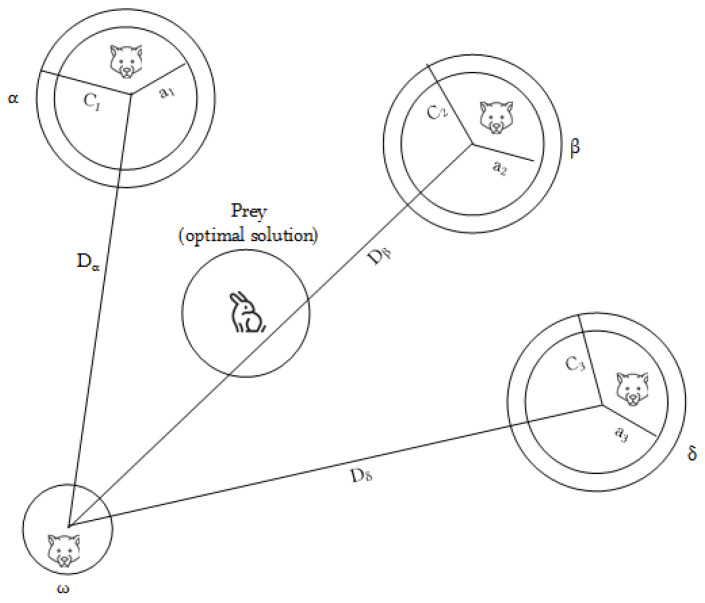
Location update of wolves.

*where $X_{\alpha }$, $X_{\beta },$ and $X_{\delta }$ represent the current position vectors of α, β and δ respectively, and X is the position of the ω. $D_{\alpha }$, $D_{\beta }$, $D_{\delta }$ indicates the relative distances between the ω and the leader wolves. Besides, $X_{1}$, $X_{2}$, $X_{3}$ are the directions and steps of the ω to the leader wolves, and X(t+1) is the newly grey wolves’ position after updating. The location update of the grey wolves is shown in Figure 1.*

**Figure 2:**
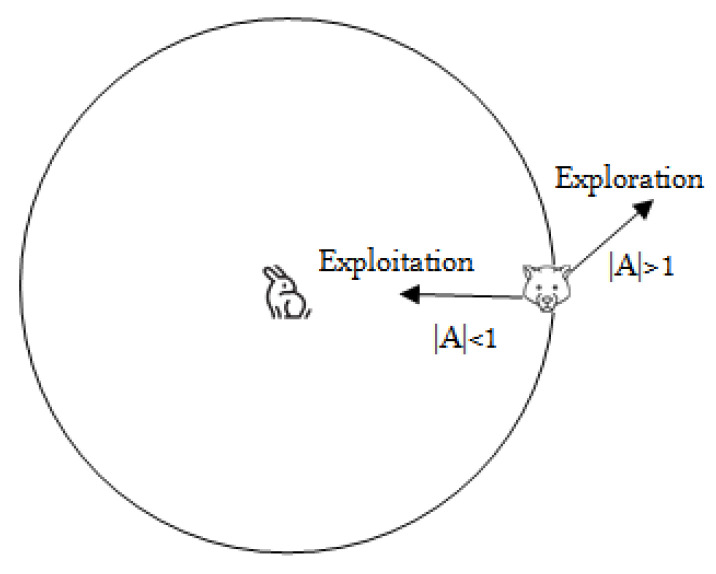
Exploration and exploitation phase.

MOGWO has two phases of exploration and exploitation. Grey wolves would be scattered throughout the space and search for prey as |A|>1, which is called the exploration phase. Otherwise, as |A|<1, grey wolves would concentrate the prey in a certain area, which is called exploitation phase as shown in Figure 2.

There are two main strategies in the MOGWO algorithm: one is the archiving strategy, and the other one is the leader selection mechanism. The archiving strategy saves the optimal wolf generated by each iteration, and the leader selection mechanism uses roulette to select the leader wolf from the archive. In the selection phase, the probability that a wolf is chosen is inversely proportional to the number of wolves in its group, as shown in Equation (7):

(7) $$P_{i}={\left(\frac{1}{N_{i}}\right)}^{c}$$

where *c* is the pressure parameter for selecting the leader (c>1), $N_{i}$ is the sum of wolves in the *i*-th group, and $P_{i}$ is the probability that the wolf is selected.

The MOGWO algorithm has the features of fewer parameters and faster convergence. However, the fast convergence sometimes does not bring benefits in the exploration phase. The linear variation of the convergence factor and the leader selection mechanism with roulette resulted in a bad balance between exploration and exploitation in the early stage. The insufficient exploration will result in the dilemma of local optimum and it is hard to find the global optimal solution. Therefore, we modify the MOGWO algorithm in Section 3.3.

### 3.3. The Modified Boltzmann-Based MOGWO

In this section, we mainly modified the MOGWO algorithm from two aspects. We improve the convergence factor first and then revise the leader selection mechanism.

#### 3.3.1. Improving the Convergence Factor

The convergence factor with linear variation is easy to get into insufficient exploration in the early stage and decreasing diversity, which will make the algorithm fall into local optimum. Therefore, we use the convergence factor based on the variation of cosine law as shown in Equation (8) to replace the linear variation:

(8) $$a=2\cos\left(\frac{t}{MaxIt}\cdot \frac{\pi }{2}\right)$$

After the modification, the convergence factor is changed nonlinearly according to the increased iterations MaxIt. Thus variation curve is a convex function. That is, at the beginning phase, since the convergence factor *a* decreases slowly, the algorithm has strong exploration ability and the diversity increases. In such time periods, the algorithm could not trap into local optimum. Entering the later phase, the convergence factor *a* decreases faster, which overcomes the flaws of the slow convergence in the later stage in traditional algorithms, and obtains the global optimal solution. Improving the convergence factor could get better effects in the exploration and exploitation process, making the algorithm more likely to get the optimal solution.

#### 3.3.2. Improving the Leader Selection Mechanism

In the MOGWO algorithm, the leader wolf is traditionally selected using a roulette-based probability mechanism. However, this method can lead to a reduction in the diversity of the wolf population, causing the algorithm to converge prematurely. To address this issue, we replace the roulette selection strategy with the Boltzmann selection strategy. The Boltzmann strategy, widely employed in machine learning and adaptive control, offers flexibility by not requiring knowledge of the objective function’s state (discrete, continuous, or divisible). Instead, the probability of selection is determined based on the estimated values of alternative solutions. Consequently, this modification enhances the likelihood of the search algorithm escaping local optima. The Boltzmann selection strategy is well used to select the leading wolf, as shown in Equation (9):

(9) $$\begin{array}{cc} \; & P_{i}=\frac{\exp(\frac{f_{i}}{T})}{\sum_{i=1}^{X_{n}}\exp\left(\frac{f_{i}}{T}\right)}\\ \; & T=T_{0}\left(0.99^{c-1}\right) \end{array}$$

where $f_{i}$ indicates the fitness value of the *i*-th grey wolf, *c* is the number of iterations, $T_{0}$ is the initial temperature, *T* refers to the current temperature, and $X_{n}$ is the number of grey wolves. The fitness value of the *i*-th wolf is inversely proportional to wolves numbers where located, i.e., $f_{i}=\frac{1}{N_{i}}$.

#### 3.3.3. MBB-MOGWO Algorithm

**Figure 3:**
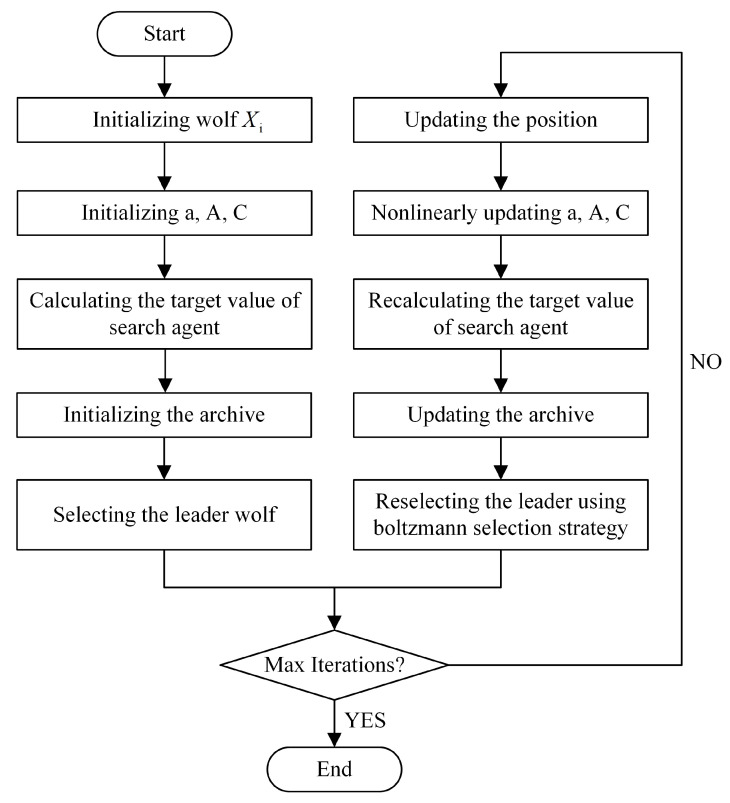
The execution flow of the MBB-MOGWO.

The execution flow of the MBB-MOGWO algorithm is presented in Figure 3.

The major operations are detailed discussed as following steps.

- Initializing the grey wolves and parameters.
- Calculating the target value of each search agent. A search agent is a wolf.
- Finding the non-dominated solutions and initializing the archive.
- Selecting the leader α from the archive and temporarily remove it; then selecting the leader β from the remaining archive and temporarily remove it too; finally selecting the leader δ and putting back the leader α, the leader β. So far, the leader wolves have been selected.
- Updating the position according to the location update equations in Section 3.2 for each search agent. During the update phase, all search agents are continually approaching the optimal solution.
- Returning the archive (and Pareto fronts should be that of all non-dominated solutions in this archive) if iterations reach to the maximum; otherwise, updating *a*, *A*, and *C*, recalculating the target values of all search agents, and updating the archive. There are four rules to follow when updating an archive:
  S1: If the new solution is dominated by solutions in original archive, it cannot enter into the archive.
  S2: If the new solution dominates at least one solution in original archive, the dominated solution is deleted as well as the new solution enters into the archive.
  S3: If the new solution has nothing to do with solutions in original archive, the new solution is archived.
  S4: If the archive is full, randomly deleting the solution in the most crowded grid, and the new solution is entered into that least crowded grid.
- Using the Boltzmann selection strategy to reselect the α, β, δ, and return to the previous step to determine if the next round of search to be continued.

The core pseudo codes of our MBB-MOGWO algorithm are provided in Algorithm 1.
**Algorithm 1** Core pseudo codes of the MBB-MOGWO**Input:** initial number of the grey wolves *n*,             the termination criteria MaxIt,             the size of archive ArcSize **Output:** the current optimal candidate solution set archive1:Initialize the grey wolves $X_{i}\left(i=1,2,...,n\right),t,MaxIt$2:$a=2\cos(\frac{t}{MaxIt}\cdot \frac{\pi }{2})$3:$X_{i}.cost$ = CalculateAgent($X_{i}.position$)4:archive = GetNonDominatedParticles(*X*, ArcSize)5:$X_{\alpha }$ = ChooseLeader(archive) and Remove $X_{\alpha }$ from the archive6:$X_{\beta }$ = ChooseLeader(archive) and Remove $X_{\beta }$ from the archive7:$X_{\delta }$ = ChooseLeader(archive) and Re-add $X_{\alpha }$, $X_{\beta }$ to the archive8:**while** (t<MaxIt) **do**9:     **for** each $X_{i}$ **do**10:     UpdatePosition($X_{i}$)11:   **end for**12:   Update *a* with $a=2\cos(\frac{t}{MaxIt}\cdot \frac{\pi }{2})$13:   Re-calculate $X_{i}.cost$ and archive14:   **if** archive is full **then**15:     Delete solutions in the most crowded grid and add new solution according to S416:   **else**17:     Update the archive according to S1–S318:   **end if**19:   $X_{\alpha }$ = ChooseLeader(archive) and Remove $X_{\alpha }$ from the archive20:   $X_{\beta }$ = ChooseLeader(archive) and Remove $X_{\beta }$ from the archive21:   $X_{\delta }$ = ChooseLeader(archive) and Re-add $X_{\alpha }$, $X_{\beta }$ to the archive22:   t=t+123:**end while**24:**return** archive

## 4. Experiments and Results Analysis

To verify whether the modified algorithm can improve the deficiencies of the traditional algorithm. We tested with the CEC 2009 and ZDT benchmark functions and compared them with the four representative algorithms.

### 4.1. Experiment Environment

The experiment configurations are shown in the Table 1.

The key parameters in the experiments are set without loss of generality. The maximum of iterations is set to 250. The number of grey wolves is set to 100. The initial temperature is set to 800. We perform experiments in the way that each algorithm tests 20 times for each benchmark function.

### 4.2. Performance Metrics

We use HV (hypervolume) [30], IGD (Reverse Generation Distance) [31], and Spread [32] as experimental indicators, which are widely used to evaluate the performance of multi-objective optimization methods, including the convergence and diversity of algorithm solutions.

HV represents the volume of the region in the target space as shown in Equation (10):

(10) $$HV\left(S,R\right)=volume\left(\overset{\left|S\right|}{\underset{i=1}{⋃}}v_{i}\right)$$

where the number of non-dominated solution sets is defined as |S|, and $v_{i}$ represents the hyper-volume computed according to the *i*-th solution in the solution set and reference point. *R* are the extreme (bounding) solutions. The larger the HV metric, the better the convergence and diversity of the algorithm solutions.

IGD indicates the average distance from every reference point to nearest solution, as shown in Equation (11):

(11) $$IGD\left(P,P^{*}\right)=\frac{\sum_{x\in P^{*}}min_{y\in P}dis\left(x,y\right)}{\left|P^{*}\right|}$$

where *P* are solutions obtained by the algorithm, $P^{*}$ are the extreme (bounding) solutions, dis(x,y) represents the Euclidean distance between point x and point y. The IGD metric serves as a comprehensive measure for evaluating both the convergence and diversity of an algorithm, providing insights into its overall accuracy. A smaller IGD value indicates improved performance of the algorithm.

Spread measures the breadth of the solutions, as shown in Equation (12):

(12) $$Spread\left(S,P\right)=\frac{d_{f}+d_{l}+\sum_{i=1}^{|S|-1}\left|d_{i}-\bar{d}\right|}{d_{f}+d_{l}+\left(|S|-1\right)\bar{d}}$$

where the Euclidean distance between consecutive solutions is indicated as $d_{i}$, and $\bar{d}$ is the average value of all $d_{i}$. The minimum Euclidean distances from solutions in *S* to the extreme (bounding) solutions of the *P* is referred as $d_{f}$ and $d_{l}$. Thus, as the Spread metric getting smaller, the spread of the solutions are surely better.

### 4.3. Results and Discussion

We use multi-objective performance metrics to evaluate the five algorithms. Each algorithm runs 20 times on the benchmark functions of UF2, UF5, UF9, ZDT2 and ZDT3. UF2 and ZDT2 are unconstrained continuous dual-objective functions, UF5 is an unconstrained discrete dual-objective function, ZDT3 is an infinitely-constrained discontinuous dual-objective function, and UF9 is a tri-objective function. We calculate the mean and standard deviation of the HV, IGD, and Spread as metrics, and the final results are shown in Table 2.

The comparative analysis reveals that our method consistently outperforms other algorithms in most cases, as indicated by superior performance metrics. We identified the optimal values for each metric across different algorithms. Across the five benchmark functions, our method consistently achieves optimal average Inverted Generational Distance (IGD) values. Furthermore, except the UF9 function, our method also attains optimal average Hypervolume (HV) values. In the case of the UF9 function, although the MOEA/D algorithm demonstrates a higher average HV, its average Spread is excessively large, indicating a limited distribution range for solutions. This suggests that the solutions obtained by the MOEA/D algorithm may lack the precision achieved by our method. However, for UF2, ZDT2, and ZDT3, although HV and IGD perform better, the spread value is large, indicating that the scalability of the solution is affected.

**Figure 4:**
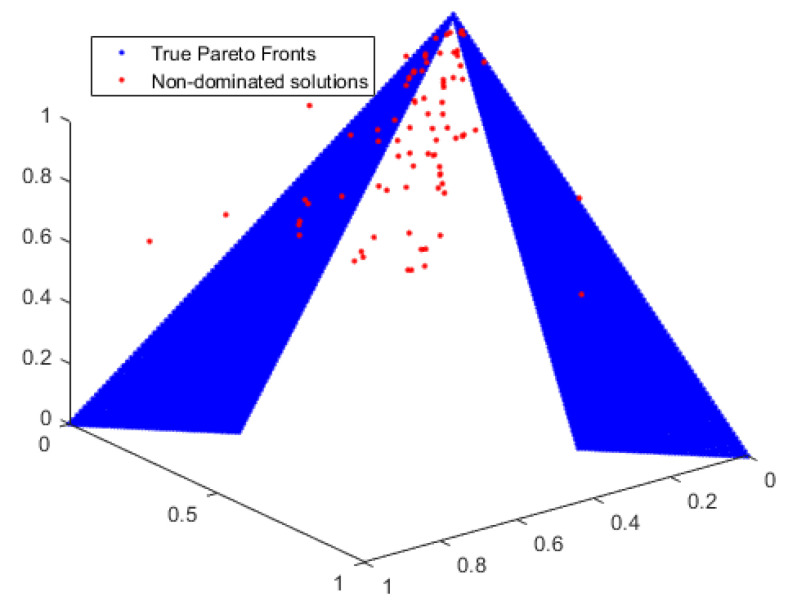
The solution coverage of MOEA/D on UF9.

**Figure 5:**
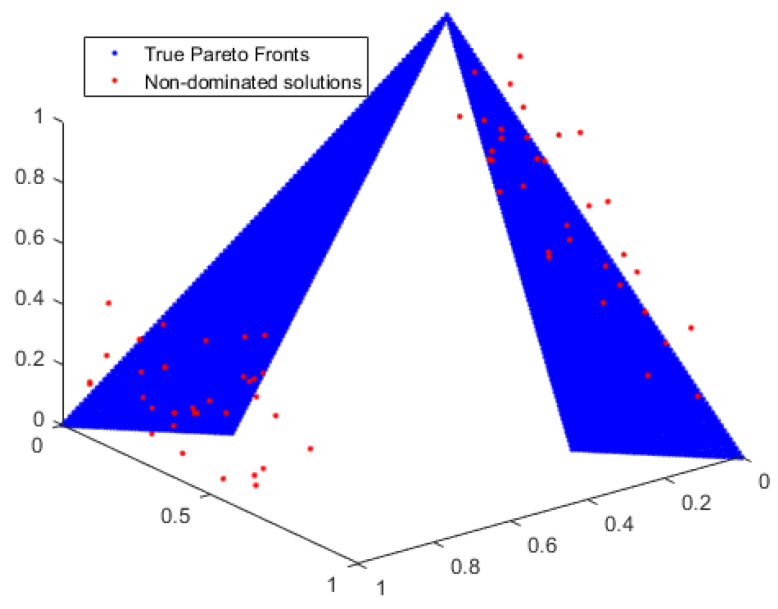
The solution coverage of MBB-MOGWO on UF9.

To demonstrate the accuracy of our work, we compare the non-dominated solutions generated by MOEA/D and MBB-MOGWO with the true Pareto fronts of UF9. We observe the coverage of the solutions to assess their accuracy. The comparison results are depicted in Figure 4 and Figure 5.

The blue dots represent the true Pareto fronts of the benchmark function UF9, and the red dots represent the non-dominated solutions obtained by the MOEA/D algorithm and MBB-MOGWO algorithm. The more the red dots fall on the blue dots, the higher the coverage of the solutions. From Figure 4 and Figure 5, we can see that the solutions obtained by our method have a wider coverage and higher accuracy.

**Figure 6:**
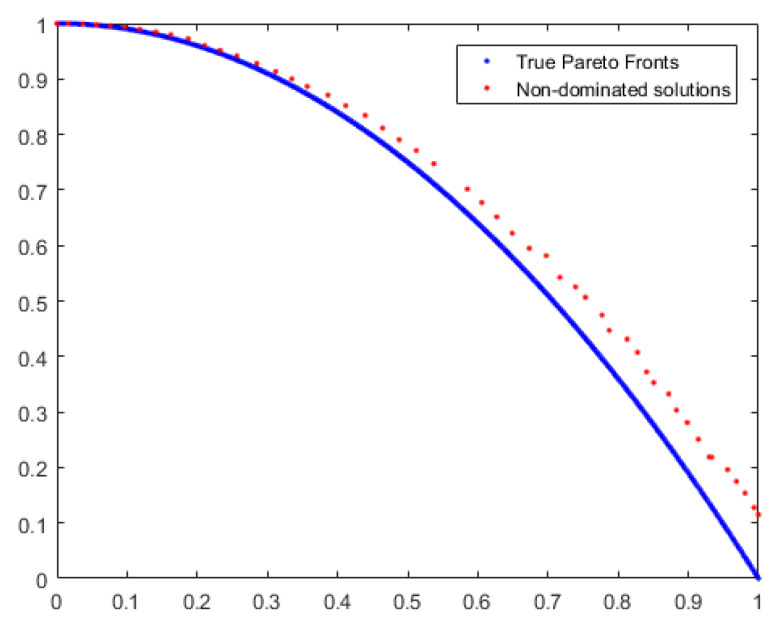
The solution coverage of MOEA/D on ZDT2.

**Figure 7:**
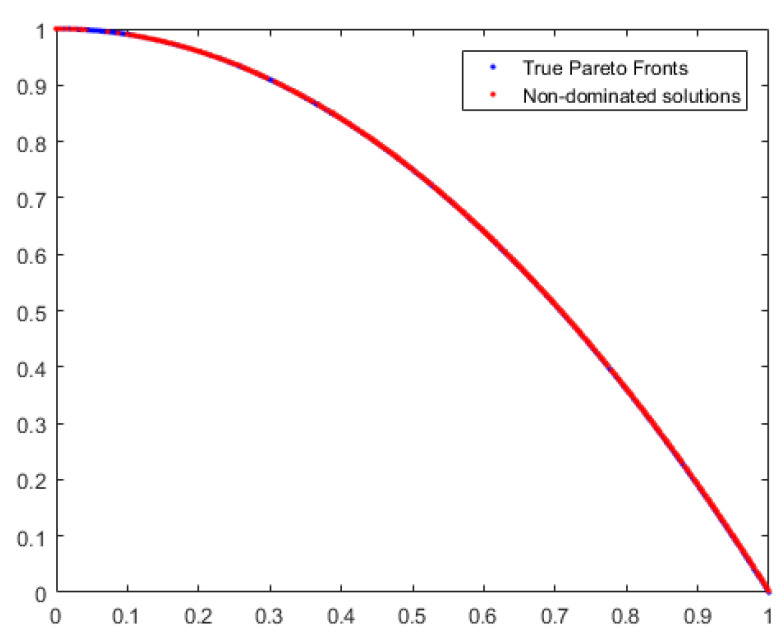
The solution coverage of MBB-MOGWO on ZDT2.

According to Table 2, MOPSO has the lowest average Spread on the UF2 function, MOEA/D has the lowest average Spread on the ZDT2 function, and NSGA-II has the lowest Spread on the ZDT3 function. But MBB-MOGWO has the optimal average IGD and average HV on these functions. On ZDT2 and ZDT3 functions, the average IGD is even two orders of magnitude lower than the above algorithms. Therefore, although the average Spread of MBB-MOGWO is not the lowest, the solution obtained is better. Below we take the ZDT2 function as an example to prove that our algorithm can get a better solution. The results are shown in Figure 6 and Figure 7.

The blue dots and red dots in the figures still represent the reference solutions and the obtained solutions, respectively. We also can see that the solutions obtained by our method have a wider coverage and higher accuracy.

Overall, by comparing the values of the multi-objective performance metrics of the five algorithms on the five benchmark functions and plotting the coverage of the solutions, we find that our method has faster convergence and diversity. Besides, the solutions obtained by our method have a wider coverage and higher accuracy.

## 5. MBB-MOGWO-Based Web Service Composition

In this section, we transform the web service composition problem into a QoS-aware multi-objective optimization problem. By optimizing metrics in $q_{G}$, the optimal solution for the web service composition can be obtained. Then how to optimize multiple metrics in $q_{G}$ is key point. Besides, we apply the modified algorithm proposed previously to the web service composition scenario and evaluate it with QWS dataset.

### 5.1. Modeling the Web Service Composition

Usually, web services have functional attribute and QoS attribute. The functional attribute refers to the functions that the web service can provide. The QoS attribute includes a series of metrics such as throughput, response time, reliability, and availability. In optimization problems within the IoT domain, these metrics and functions are employed to gauge critical attributes such as the cost of resource scheduling, system stability, and real-time performance associated with sensors. When users select different web services to combine, the higher QoS means the better service quality and the better composition solution, with the premise that the function is satisfied. Since QoS has multiple metrics to measure the quality of service, we can abstract the web service composition problem into a QoS-aware multi-objective optimization problem. Below we abstract the web service composition and give some relative definitions.

**Definition** **6** (QoS multi-tuple)**.** *QoS means the quality of a web service. The QoS multi-tuple is represented by the vector $Q:=(q_{1},q_{2},...,q_{m})$, where m represents QoS has a total of m metrics, $q_{i}$ represents the value of the i-th metric in m, i∈[1,m].*

*QoS attributes often have two categories. One aspect refers to the positive attributes, i.e., the bigger attribute values cause the better QoS, such as throughput, availability or reliability, etc. The other aspect refers to the negative attribute, i.e., the bigger attribute values cause the worse QoS, such as price or response time, etc.*

**Definition** **7** (Abstract web service)**.** *We define the abstract web service as a two-tuple T:=(Q,Seq), where Q means the QoS of specific web service and Seq means the execution relationship between the web services.*

**Figure 8:**
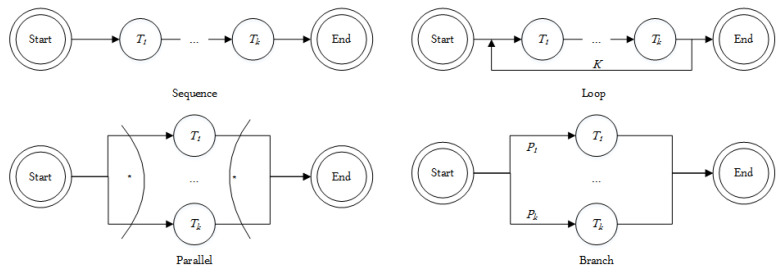
Execution relationships of web services.

*There are four execution relationships between the web services: sequence, loop, parallel, and branch, as shown in Figure 8.*

*The sequence relationship means that all subtasks are executed one by one; the parallel relationship means that all subtasks are executed at the same time, which does not interfere with each other; the loop relationship refers to the subtasks being executed iteratively; the branch relationship means that only one branch will be selected for execution.*

**Definition** **8** (Abstract web service composition)**.** *We define the abstract web service composition as a tri-tuple $S:=(s,q_{G},w)$, where $s=(T_{1},T_{2},...,T_{k})$, i.e., a web service composition s consists of k different web services; $q_{G}=\left(q_{g1},q_{g2},...,q_{gm}\right)$ represents the global QoS of the web service composition; $q_{gi}$ represents the value of the i-th metric in m global QoS metrics, i∈[1,m]; $w=(w_{1},w_{2},...,w_{m})$ represents the weight of each QoS metric, and $w_{1}+w_{2}+...+w_{h}=1$.*

*Through the above abstract description, we transform the web service composition problem into a QoS-aware multi-objective optimization problem. By measuring the $q_{G}$ of the web service composition, we can judge the pros and cons of the combination solutions. The $q_{G}$ is a combination of the $Q:=(q_{1},q_{2},...,q_{m})$ of each web service in the composition. Different execution sequences have different aggregation equations to calculate the $q_{G}$ which consists of $q_{gi}$. As shown in Table 3, $q_{gi},i\in \left[1,6\right]$ represents the six QoS metrics, and $q_{G}=\left(q_{g1},q_{g2},...,q_{g6}\right)$.*

### 5.2. Application of MBB-MOGWO on Web Service Composition

**Figure 9:**
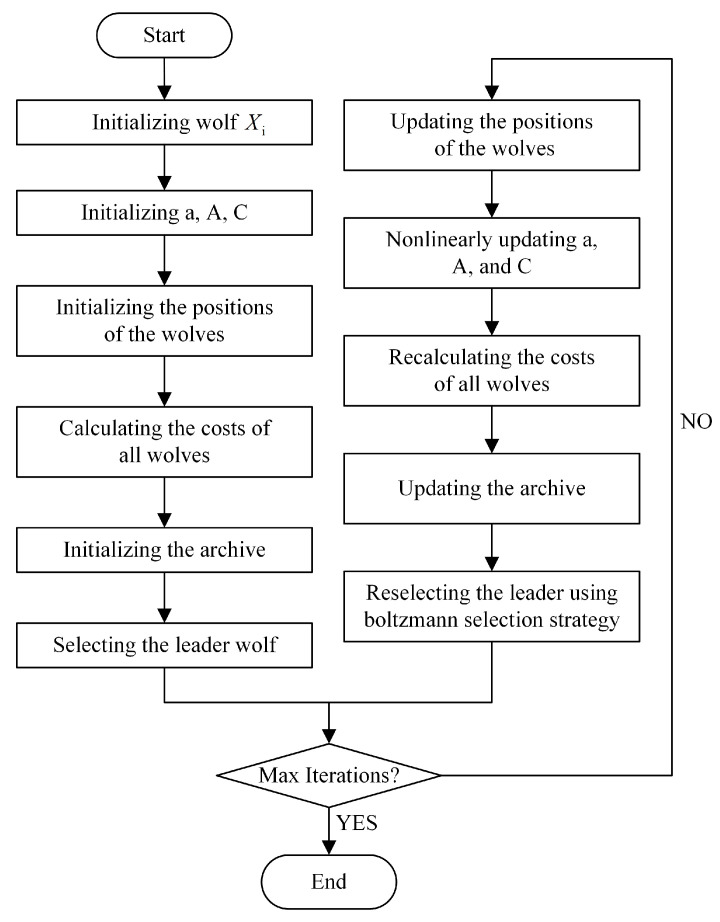
The execution flow of MBB-MOGWO-based service composition.

The execution flow of MBB-MOGWO-based web service composition is shown in Figure 9.

The key content of combining the MBB-MOGWO with the web service composition mainly includes initializing the positions of the wolves, calculating the costs of all wolves, and updating the positions of the wolves. In these three parts, we have adopted encoding, fitness function and position update strategy, which make our method more reasonable. We will discuss the three parts as follows.

#### 5.2.1. Encoding

In the scenario of web service composition, the global QoS depends on the execution relationship between each subtask. This paper considers the sequential workflow. We assume that each wolf represents a solution to the web service composition. Each wolf has a position vector and a cost vector. The dimension of the wolf’s position is the number of required subtasks. The range of each dimension is the candidate services for the subtask. We need to select one of the candidate services for every subtask and save the global QoS as the cost vector of Wolf. Finally, by comparing the cost, the MBB-MOGWO algorithm updates the archive and obtains the optimal web service composition.

In order to make the algorithm more suitable for web service composition problem, we use integer coding to indicate which candidate service is selected for each subtask. For example, $S:=(s,q_{G},w)$, where $s=(T_{1,3},T_{2,4},T_{3,1},T_{4,2})$. That is, the web service composition has four subtasks, subtask 1 selects the candidate service 3, subtask 2 selects the candidate service 4, and so on. *s* is the position vector of a grey wolf, and *S* represents each grey wolf.

#### 5.2.2. Fitness Function

In the optimization process, we need to judge the adaptability of each wolf through the fitness function, and retain the wolves with higher fitness, so that the wolves continue to approach the optimal solution. Since QoS has two types of attributes, i.e., positive attribute and negative attribute, we transform the web service composition problem into a bi-objective problem that optimizes the positive attribute and the negative attribute, shown in Formula (13):

(13) $$\begin{array}{cc} \; & minP\left(x\right)=-(T\left(x\right)\times w_{1}+A\left(x\right)\times w_{2}+S\left(x\right)\times w_{3},...)\\ \; & minN\left(x\right)=(t\left(x\right)\times w_{4}+l\left(x\right)\times w_{5}+p\left(x\right)\times w_{6},...) \end{array}$$

where *P* represents the positive attribute, *T*, *A*, and *S* represent throughput, availability, and success rate respectively; *N* represents the negative attribute, *t*, *l*, and *p* represent response time, latency, and price respectively. $w=(w_{1},w_{2},w_{3},w_{4},w_{5},w_{6},...)$ is the weight of each QoS metric. The smaller the values of *P* and *N*, the higher the fitness of wolf, that is, the better the solution of the web service composition.

In the fitness function, each QoS metric (such as T(x)) is calculated by the aggregation equation in Section 5.1. Since the values of the different metrics have large differences, normalization processing is required before they are used. Assuming $q_{h}$ represents the *h*-th metric of the QoS attribute, we use Equation (14) to normalize all the metrics:

(14) $$q_{h}=\left\{\begin{array}{cc} \frac{q_{h}-q_{h}^{min}}{q_{h}^{max}-q_{h}^{min}} & q_{h}\;\mathrm{is}\;\mathrm{the}\;\mathrm{positive}\;\mathrm{metric}\;\\ \frac{q_{h}^{max}-q_{h}}{q_{h}^{max}-q_{h}^{min}} & q_{h}\;\mathrm{is}\;\mathrm{the}\;\mathrm{negative}\;\mathrm{metric}\; \end{array}\right.$$

after normalization, all values are stipulated between [0,1].

#### 5.2.3. Position Update

In MBB-MOGWO algorithm, the position information of the wolves’ changes within a continuous range, and the calculation rules involved in the algorithm are also for continuous variables. However, as we presented in previous subsection, the candidate service for each subtask is a discrete number based on integers, so we need to discretize the continuous variables.

There are usually three main discretization strategies, probability processing, operator redefinition, and direct conversion. However, the method of probabilistic processing has too few application scenarios, and operator redefinition has higher complexity. Therefore, we use the direct conversion method to discretize the position information of the wolves. After each position is updated, we replace the actual position of the grey wolf with the nearest value from the actual position in the discrete domain. Despite there may be cases where lots of continuous variables point to the same discrete variable, the calculation results show that in the high dimensional optimization problem, after the discretization processing, the algorithm still has high stability and does not fall into local optimum.

### 5.3. Experiments and Results Analysis

According to the NFL theorem [33], no perfect optimization methods exist to solve all kinds of optimization issues. The superiority of the optimizer to a type of problems is not necessarily useful for another type of problems. So we need to make an evaluation of our modified method. We select the QWS dataset that is commonly used in web service composition problems to evaluate our method. The QWS dataset contains 2507 real web service data [15], and a total of 9 QoS metrics are counted. The 9 QoS metrics and their descriptions are shown in Table 4.

Among these QoS metrics, we have selected six more important metrics, which are availability, reliability, throughput, response time, success rate and latency. The response time and latency are negative attributes, and the rests are positive attributes. We select 2500 data in QWS dataset to conduct experiments. We assume that a web service composition consists of 10 subtasks, and each subtask has 250 candidate services.

In the experiments, we compare the MBB-MOGWO algorithm with NSGA-II algorithm, MOEA/D algorithm, MOPSO algorithm and MOGWO algorithm. The experiment environment is the same as Section 4.1 and the key parameters are configured as follows. The maximum of iterations is set to 100. The number of grey wolves is set to 100. Initial temperature is set to 600. We perform experiments in the way that each algorithm tests 20 times.

To evaluate our method, we calculated the best, worst, average, and standard deviation of HV in 20 web service composition experiments for each algorithm, which is shown in Table 5. The analysis demonstrates that in both the best and worst cases, the HV values of MBB-MOGWO consistently surpass those of other algorithms, with the average result being the highest. This suggests that the MBB-MOGWO algorithm exhibits superior convergence and diversity in addressing web service composition problems. Notably, among the five algorithms, MBB-MOGWO boasts the lowest standard deviation of HV, indicating enhanced stability compared to its counterparts.

**Figure 10:**
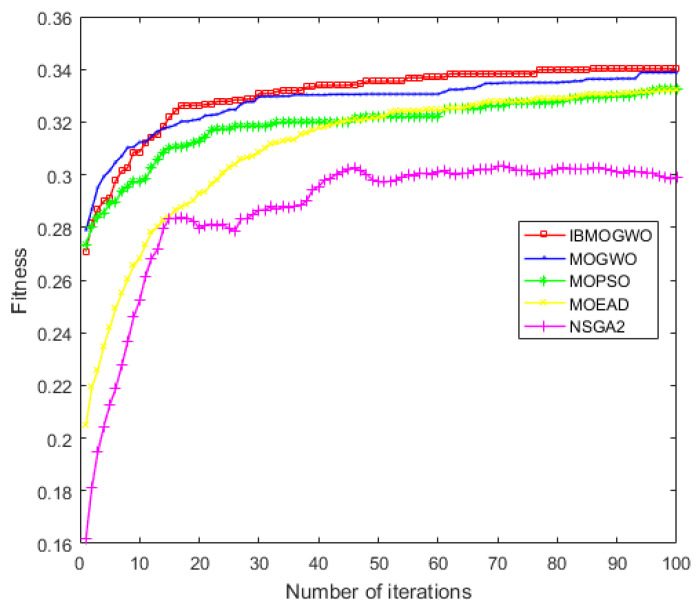
Fitness of the positive attribute.

**Figure 11:**
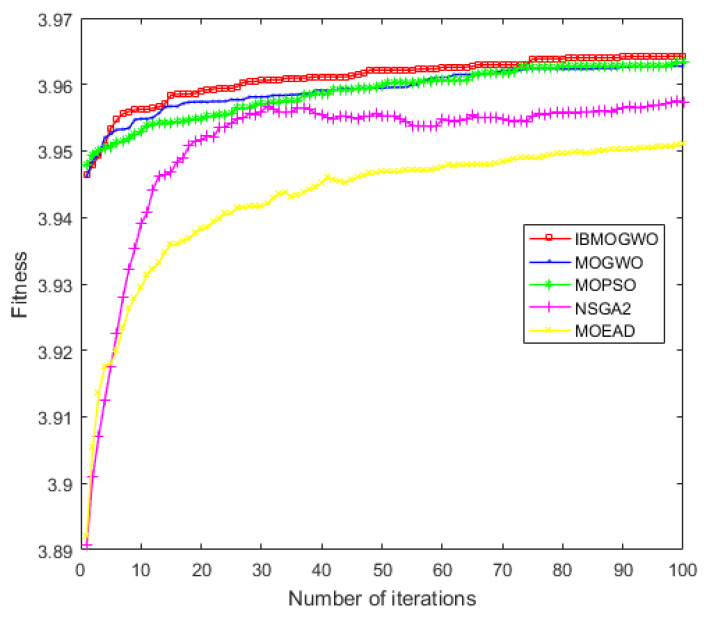
Fitness of the negative attribute.

To further illustrate the advantages of the MBB-MOGWO algorithm, we averaged the experimental results of 20 tests for each algorithm and plotted the change trend of the fitness values, which shown in Figure 10 and Figure 11.

The abscissa represents iterations, and the ordinate represents the fitness values of the positive/negative attribute. From the figures, we can see that the fitness values of all methods increased rapidly at the beginning of the experiment. With iteration increases, the trend of fitness values tends to be stable. Finally, the fitness of our method is higher than the baseline algorithms. That is, the QoS of the web service composition found by our method is better.

Through the evaluation, we find that the MBB-MOGWO algorithm shows better performance on the web service composition problem, which has fast convergence speed and diversity. By balancing the exploration and exploitation phases, it does not easily fall into a local optimum, which improves the accuracy of the solution. So the MBB-MOGWO algorithm is more conducive to finding a better quality web service composition.

## 6. Conclusions

In this paper, MBB-MOGWO is proposed to make novel improvements towards MOGWO. MBB-MOGWO proposes the convergence factor used in the position update of a wolf as the variation of cosine law and introduces the Boltzmann selection strategy to maintain a better balance in the exploration and exploitation phase. To verify our method, we use multi-objective benchmark functions to make the evaluation and compare our method with four representative algorithms. By comparing the multi-objective performance metrics IGD, HV, and Spread, we demonstrate that the MBB-MOGWO algorithm had better convergence and diversity. Besides by comparing the coverage of the Pareto front, we find that the solution found by our method had higher precision and wider coverage. Then we apply the MBB-MOGWO algorithm to the actual scenario, i.e., computing optimal web service composition. We use 2500 real data records in the QWS standard dataset for the experiment and compare our method also with four representative algorithms. By comparing the changes of the fitness and performance metric HV, the experiment results show that the solutions found via the MBB-MOGWO algorithm have higher fitness and better service quality. Our future research entails the application of the MBB-MOGWO algorithm to various real-world tasks, including IoT resource scheduling and energy optimization associated with sensors. This endeavor is aimed at substantiating the practical viability and scalability of the algorithm in diverse and complex scenarios.

## Figures and Tables

**Table 1:** Configurations in experiment.

Environment	Configurations
CPU	2 core 2.60 GHz
Memory	12 GB
Disk	1T
OS	Windows 8.1
Software	Matlab R2016a

**Table 2:** Mean and standard deviation of the performance metrics.

Algorithm	Metrics	UF2	UF5	UF9	ZDT2	ZDT3
	HV	0.3091 ± 0.0460	0.0024 ± 0.0104	0.0206 ± 0.0475	0.0430 ± 0.0466	0.5158 ± 0.0724
NSGA-II	IGD	0.0078 ± 0.0012	0.2196 ± 0.0580	0.0090 ± 0.0030	0.0269 ± 0.0162	0.0193 ± 0.0030
	Spread	1.0888 ± 0.1522	1.2265 ± 0.1032	0.9944 ± 0.0909	0.8030 ± 0.1767	**0.8542 ± 0.0329**
	HV	0.6045 ± 0.0062	0 ± 0	**0.3345 ± 0.0658**	0.2776 ± 0.0137	0.7283 ± 0.0179
MOEA/D	IGD	0.0019 ± 2.7069 × $10^{-4}$	0.4188 ± 0.1165	0.0034 ± 4.4090 × $10^{-4}$	0.0018 ± 6.3647 × $10^{-4}$	0.0031 ± 0.0017
	Spread	0.7475 ± 0.1646	1.0695 ± 0.1167	1.0511 ± 0.0911	**0.3438 ± 0.1153**	1.0077 ± 0.0663
	HV	0.6024 ± 0.0103	0 ± 0	0.2064 ± 0.0695	0.1092 ± 0.0316	0.3316 ± 0.0285
MOPSO	IGD	0.0026 ± 0.0011	0.2998 ± 0.0827	0.0042 ± 5.9027 × $10^{-4}$	0.0136 ± 0.0022	0.0250 ± 0.0014
	Spread	**0.7375 ± 0.0674**	1.0074 ± 0.0773	0.7592 ± 0.0792	0.9352 ± 0.0763	1.0409 ± 0.0293
	HV	0.6055 ± 0.0092	0.0011 ± 0.0034	0.3243 ± 0.0995	0.3310 ± 0.0016	0.7766 ± 0.0026
MOGWO	IGD	0.0017 ± 2.4507 × $10^{-4}$	0.1923 ± 0.0536	0.0029 ± 7.8542 × $10^{-4}$	7.8441 × $10^{-5}$ ± 6.7862 × $10^{-6}$	2.1368 × $10^{-4}$ ± 6.1963 × $10^{-5}$
	Spread	0.8535 ± 0.0745	0.9000 ± 0.2035	0.7686 ± 0.0643	0.8255 ± 0.0429	1.1257 ± 0.0425
	HV	**0.6074 ± 0.0058**	**0.0084 ± 0.0376**	0.3322 ± 0.0769	**0.3316 ± 0.0017**	**0.7786 ± 0.0019**
MBB-MOGWO	IGD	**0.0016 ± 1.2076 × $10^{-4}$**	**0.1864 ± 0.0542**	**0.0029 ± 6.7108 × $10^{-4}$**	**7.3189 × $10^{-5}$ ± 9.5475 × $10^{-6}$**	**1.1580 × $10^{-4}$ ± 2.4036 × $10^{-5}$**
	Spread	0.8528 ± 0.0814	**0.8699 ± 0.1373**	**0.7368 ± 0.0684**	0.7022 ± 0.0270	0.9885 ± 0.0343

The bold format indicates the optimal results in the table.

**Table 3:** Aggregation equations.

QoS Metric	Sequence	Branch (n Selected)	Parallel	Loop (k Times)
Response Time	$\sum_{i=1}^{k}q_{i}$	$max_{i=1}^{n}\;q_{i}$	$max_{i=1}^{k}\;q_{i}$	$k*q_{i}$
Reliability	$\prod_{i=1}^{k}q_{i}$	$\prod_{i=1}^{n}q_{i}$	$\prod_{i=1}^{k}q_{i}$	${\left(q_{i}\right)}^{k}$
Availability	$\prod_{i=1}^{k}q_{i}$	$\prod_{i=1}^{n}q_{i}$	$\prod_{i=1}^{k}q_{i}$	${\left(q_{i}\right)}^{k}$
Throughput	$min_{i=1}^{k}\;q_{i}$	$min_{i=1}^{n}\;q_{i}$	$min_{i=1}^{k}\;q_{i}$	$q_{i}$
Latency	$\sum_{i=1}^{k}q_{i}$	$max_{i=1}^{n}\;q_{i}$	$max_{i=1}^{k}\;q_{i}$	$k*q_{i}$
Success Rate	$\prod_{i=1}^{k}q_{i}$	$\prod_{i=1}^{n}q_{i}$	$\prod_{i=1}^{k}q_{i}$	${\left(q_{i}\right)}^{k}$

**Table 4:** The QoS metrics in QWS dataset.

ID	QoS Metrics	Description	Units
1	Response Time	from request sent till response received	ms
2	Availability	ratio of successful to total invocations	%
3	Throughput	sum of invocations for a period of time	b/s
4	Success Rate	ratio of response number to request number	%
5	Reliability	ratio of error to total messages	%
6	Compliance	conformance percentage between a specific WSDL and WSDL specification	%
7	Best Practices	compliance percentage between a specific web service and WS-I Basic Profile	%
8	Latency	time cost of server processing specific request	ms
9	Documentation	Measurement for documenting WSDL	%

**Table 5:** Comparison results of the HV.

HV	NSGA-II	MOEA/D	MOPSO	MOGWO	MBB-MOGWO
Worst	0	0	0.2500	0.3200	0.3600
Best	0.5700	0.7300	0.9000	0.9300	0.9500
Average	0.2980	0.2865	0.5330	0.5885	0.6305
STD. Dev.	0.1591	0.2314	0.1880	0.1566	0.1514

## Data Availability

Data are contained within the article.
